# The Australasian diabetes data network (ADDN): first steps towards a national database resource

**DOI:** 10.1186/1687-9856-2015-S1-O35

**Published:** 2015-04-28

**Authors:** Helen Phelan, Kim Donaghue, Fergus Cameron, Helen Clapin, Andrew Cotterill, Jenny Couper, Maria Craig, Elizabeth Davis, Craig Jefferies, Elaine Tham, Timothy Jones

**Affiliations:** 1 John Hunter Children’s Hospital, Newcastle, NSW, Australia; 2 Children’s Hospital at Westmead, Sydney, NSW, Australia; 3 Royal Children’s Hospital, Melbourne, VIC, Australia; 4 Princess Margaret Hospital, Perth, WA, Australia; 5 Mater Children’s Hospital, Brisbane, QLD, Australia; 6 Women’s and Children’s Hospital, Adelaide, SA, Australia; 7 Starship Children’s Hospital, Auckland, New Zealand; 8 Telethon Kids Institute, Perth, WA, Australia

## 

The Australasian Diabetes Data Network (ADDN) is a collaboration that aims to collect and centrally collate a suite of patient data across Australia and New Zealand that will be available to all diabetes researchers. ADDN will collect information prospectively, facilitating data sharing and building capacity in clinical service delivery and investigation. The central database will provide a research resource, facilitate study recruitment and be an ongoing source of data to enable benchmarking against national outcomes. By April 2015 ADDN will be populated with over 4,000 participants presenting an opportunity to analyse national data for the first time. To date ADDN holds the data of 1136 children and adolescents. Of the T1D participants (n=1064), males and females were equally represented, with 3% (35) aged <5, 19% (200) aged 5-10, 37%( 396) aged 10-15, 38%( 399) aged 15-20 and 3%( 34) >20 years of age. A total of 10% were treated with twice daily injections, 42% with Multiple Daily Injections and 47% with continuous subcutaneous insulin infusion (CSII). The TID group had a median HbA1c of 8% (64 nmol/mol) with 31% (330) <7.5% (58.5 nmol/mol), 42% (447) 7.5-9.0 (58.5-74.9 nmol/mol) and 27%(287) >9.0% (74.9 nmol/mol). From these preliminary results it is clear that a large proportion of participants are not meeting recommended glycaemic targets[1] despite the high uptake of intensified insulin therapy. Further analysis of the ADDN dataset is needed to understand the influence of different practices and therapies for T1D used in Australian youth, and the clinical and demographic predictors of glycaemic outcomes. This research and resource will have major implications for clinical investigation in Australasia and will provide an evidence base to inform health policy.

On behalf of the ADDN Study Group
